# Corrigendum: Preparing correctional settings for the next pandemic: a modeling study of COVID-19 outbreaks in two high-income countries

**DOI:** 10.3389/fpubh.2024.1478843

**Published:** 2024-12-03

**Authors:** Jisoo A. Kwon, Neil A. Bretaña, Nadine Kronfli, Camille Dussault, Luke Grant, Jennifer Galouzis, Wendy Hoey, James Blogg, Andrew R. Lloyd, Richard T. Gray

**Affiliations:** ^1^Kirby Institute, UNSW Sydney, Sydney, NSW, Australia; ^2^University of South Australia, Adelaide, SA, Australia; ^3^Research Institute of the McGill University Health Centre, Montreal, QC, Canada; ^4^McGill University Health Centre, Montreal, QC, Canada; ^5^Corrective Services NSW, Sydney, NSW, Australia; ^6^Justice Health Forensic Mental Health Network NSW, Sydney, NSW, Australia

**Keywords:** COVID-19, SARS-CoV-2, modeling, prison, Australia, Canada

In the published article, there was an error in the **Ethics statement**. The statement in the sections **Methods** and **Ethics statement** was incorrect. The previous sentence stated:

“No additional approval from the McGill University Health Center Research Ethics Board was necessary for the Canadian dataset as it was publicly available.”

The corrected sentence appears below:

“No additional approval from the McGill University Health Center Research Ethics Board was necessary.”

In the published article, there was an error in the **References**. One reference (53) was cited by error and was removed. The remaining references have been renumbered accordingly.

In the published article, there was an error in the **Author disclaimer**. The statement was incomplete and should mention the Ministère de la Sécurité publique, Canada. The corrected statement appears below:

