# Supplementary material for: Preparing correctional settings for the next pandemic: a modeling study of COVID-19 outbreaks in two high-income countries
Source: Front Public Health. 2024 Mar 15;12:1279572. doi: 10.3389/fpubh.2024.1279572 (PMC10978752; doi:10.3389/fpubh.2024.1279572)
Supplement: Supplementary file 1 [file Data_Sheet_1.docx]

Preparing correctional settings for the next pandemic: a modelling study of COVID-19 outbreaks in two high-income countries

**Supplementary material**

**Figure A.1: Number of infections (inmates, omicron variant) with vaccination scenarios following the entry of one infected inmate (black line: baseline line (50% vaccination of inmates) with NPIs, red line: 50% vaccination of inmates without NPIs, blue line: 100% inmate vaccination of inmates without NPIs, and yellow line: 100% vaccination of inmates with NPIs); (A) NSW, Australia and (B) Quebec, Canada**

| (A) | 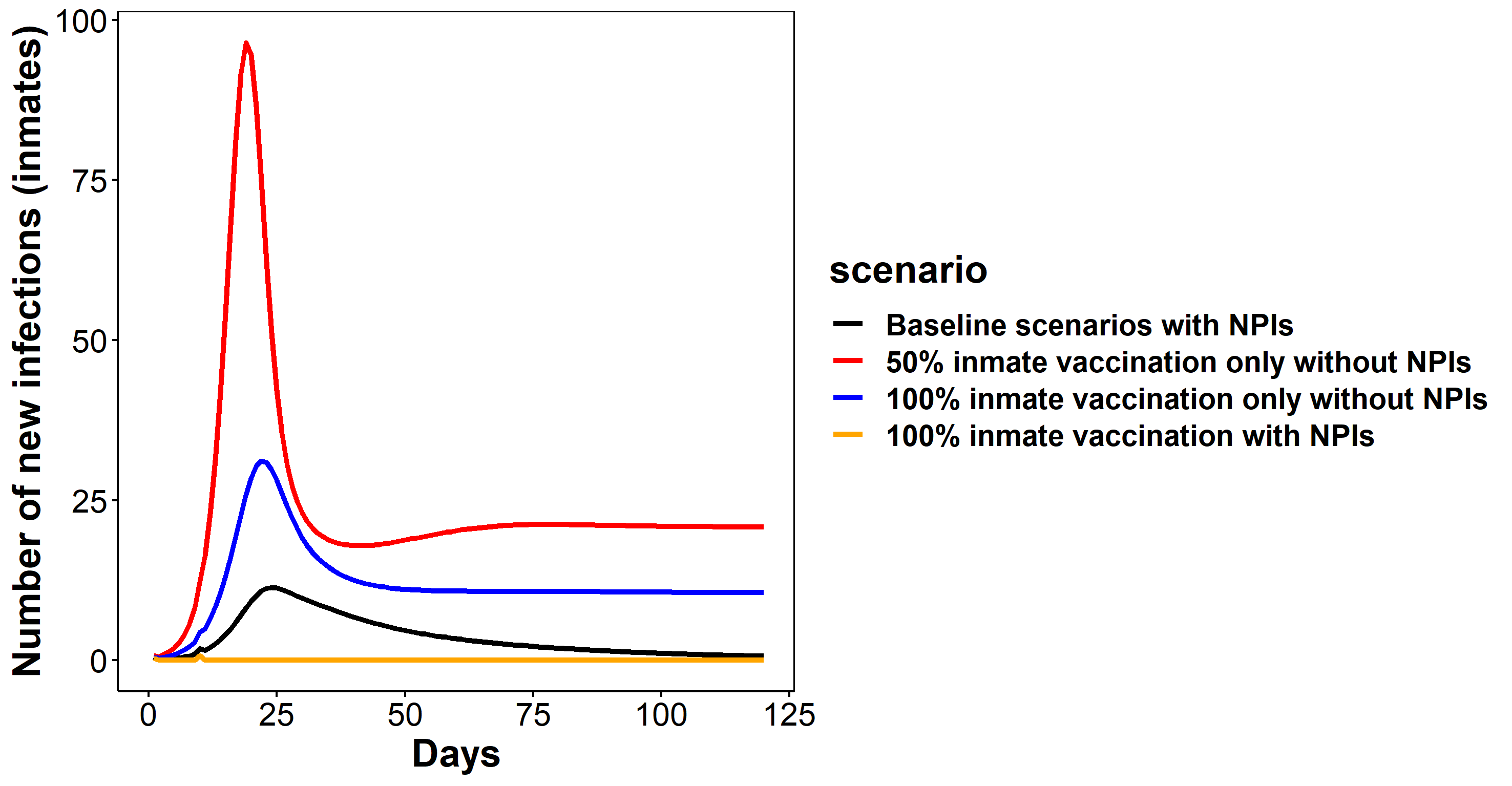 |
| --- | --- |
| (B) | 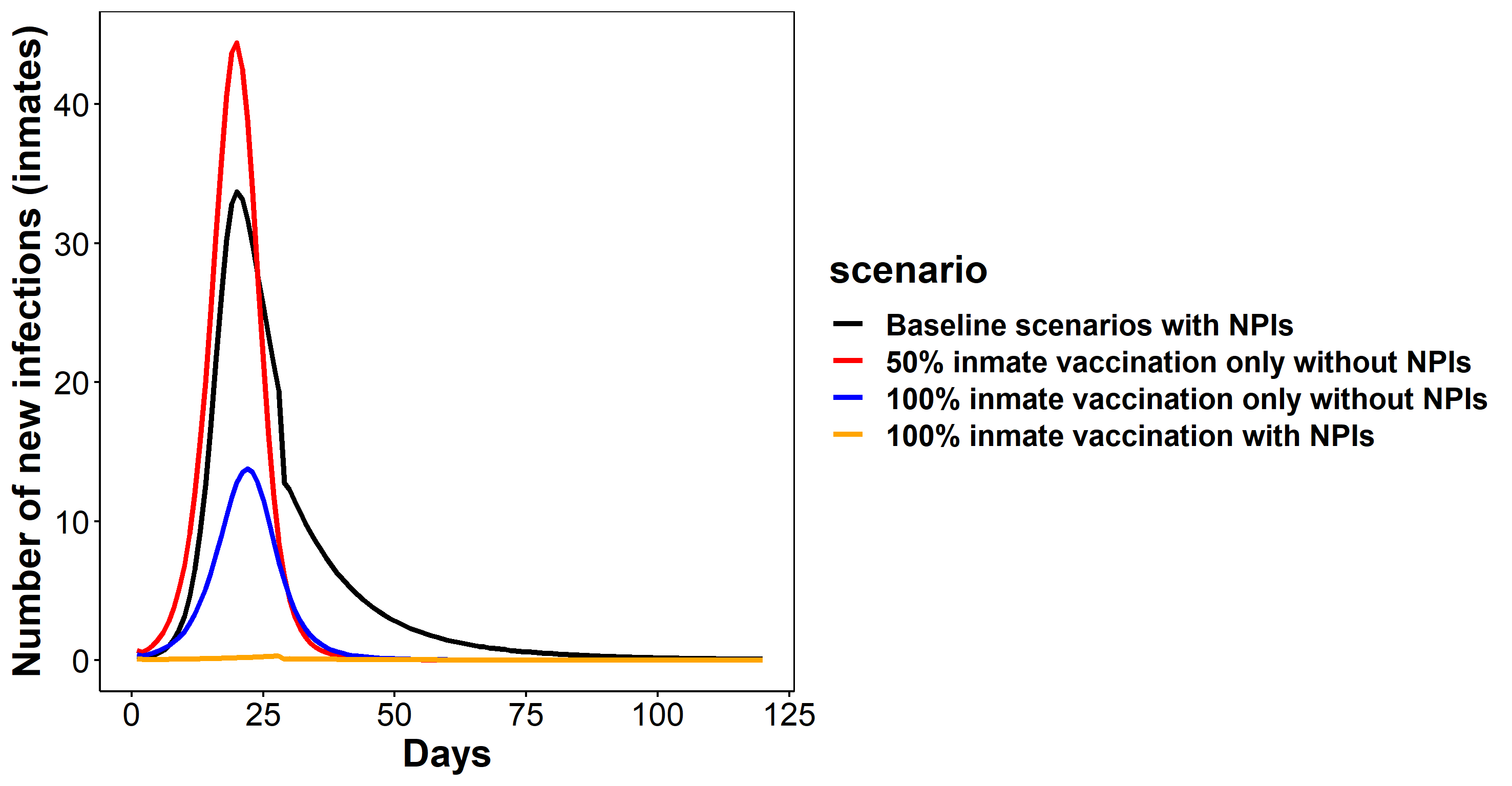 |

**Figure A.2: Number of infections (inmates, delta variant) with vaccination and NPIs scenarios following the entry of one infected inmate (black line: 50% vaccination of inmates without NPIs, red line: 100% vaccination of inmates without NPIs, blue line: 50% inmate vaccination of inmates with deferral/early release, yellow line: 50% inmate vaccination of inmates with PPE, green line: 50% inmate vaccination of inmates with quarantine, and purple line: 50% vaccination of inmates with isolation); (A) NSW, Australia and (B) Quebec, Canada**

| (A) | 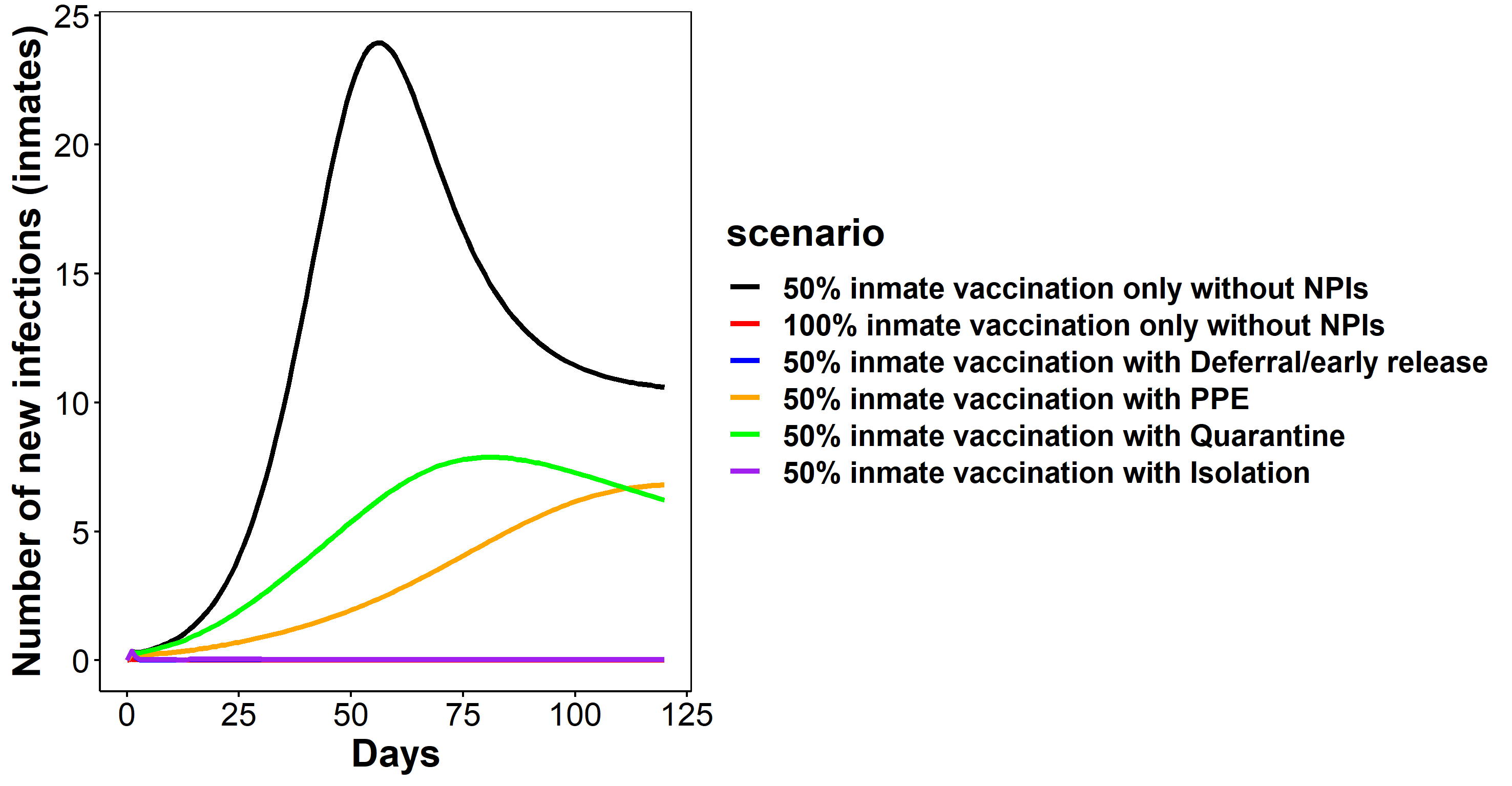 |
| --- | --- |
| (B) | 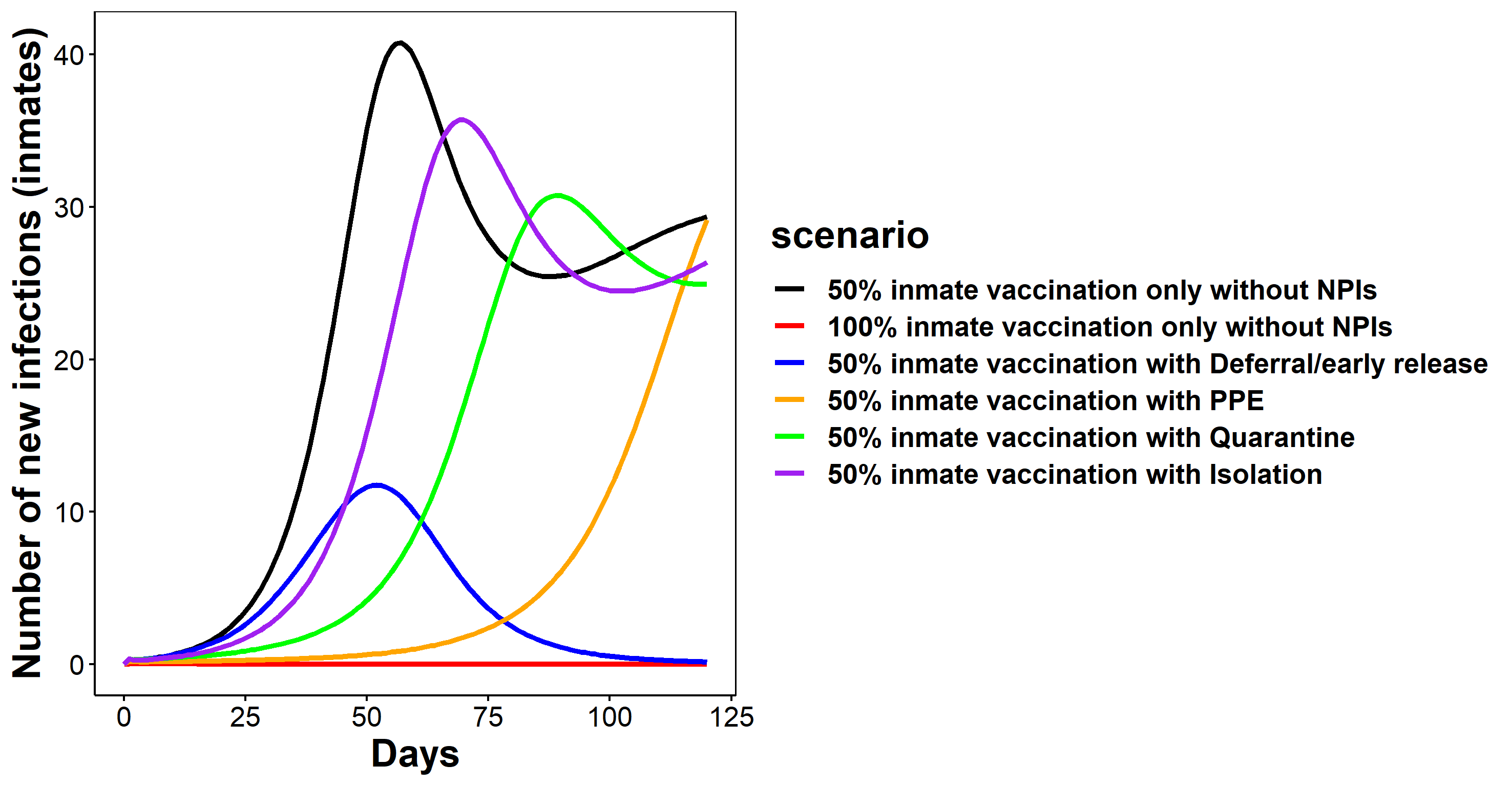 |

**Figure A.3: Number of staff available to work following the entry of one infected inmate (delta variant, blue line: vaccinating 100% staff only without NPIs and red line: no vaccination for staff without NPIs; (A) NSW, Australia and (B) Quebec, Canada, if an outbreak was initiated by an inmate.**

| (A) | 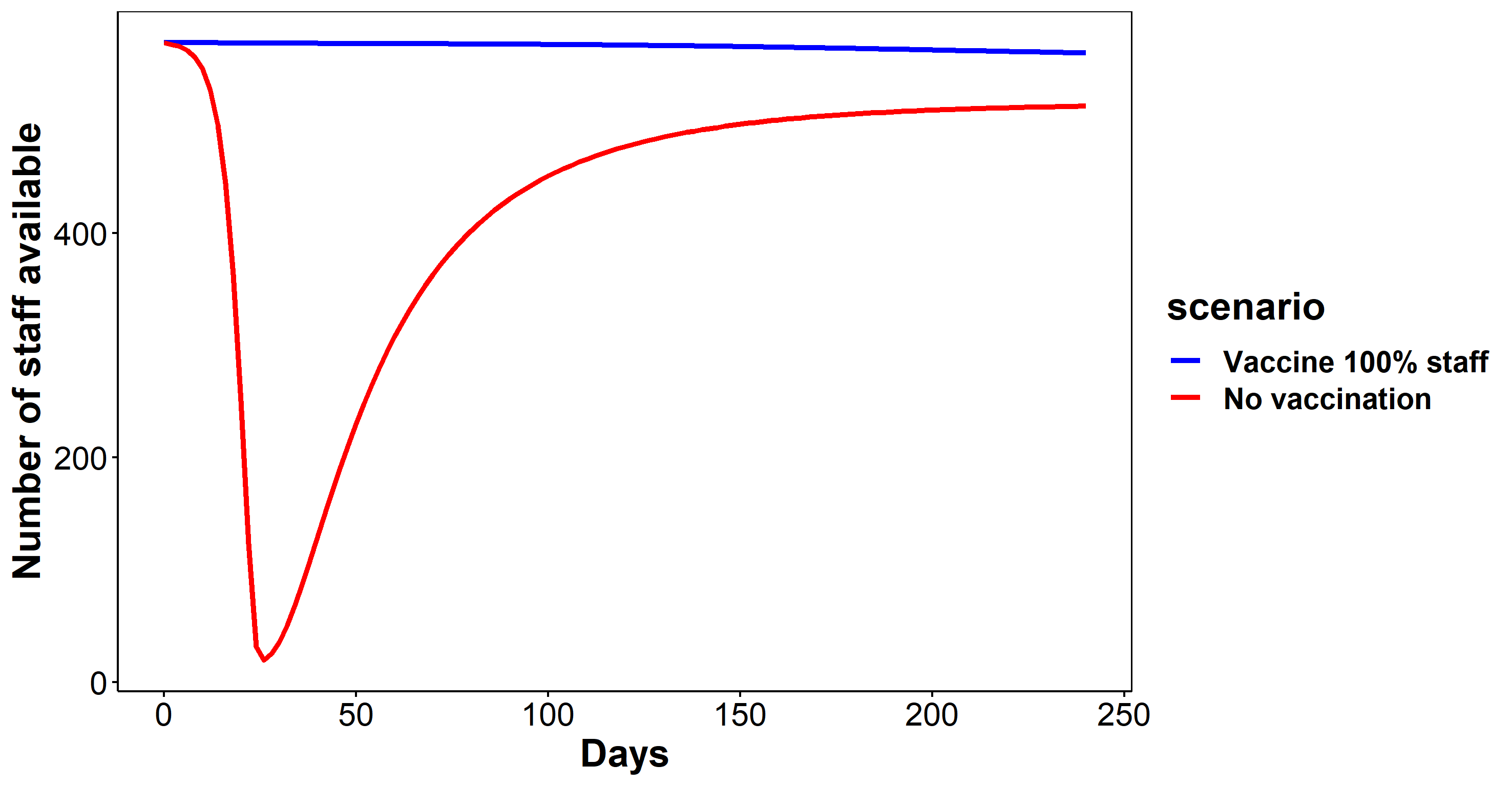 |
| --- | --- |
| (B) | 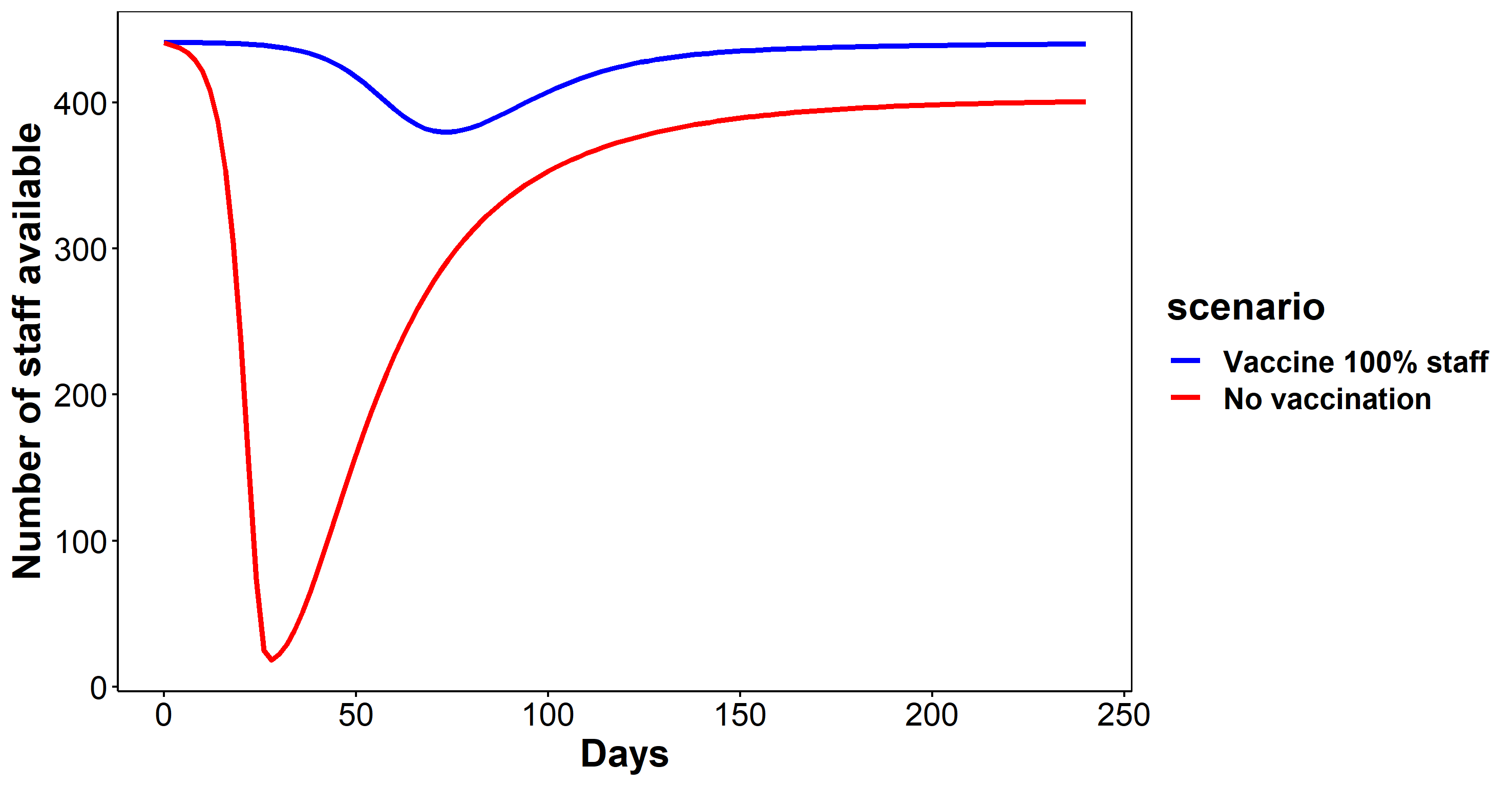 |

**Figure A.4: Tornado plot of partial rank correlation coefficients for the COVID_19 cumulative infections (inmates and staff) with the baseline scenario: (A) NSW, Australia and (B) Quebec, Canada**

| (A) | 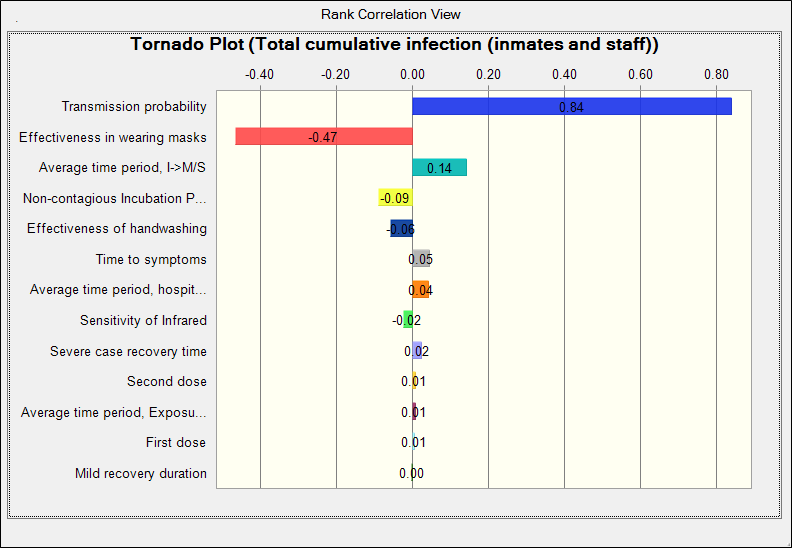 |
| --- | --- |
| (B) | 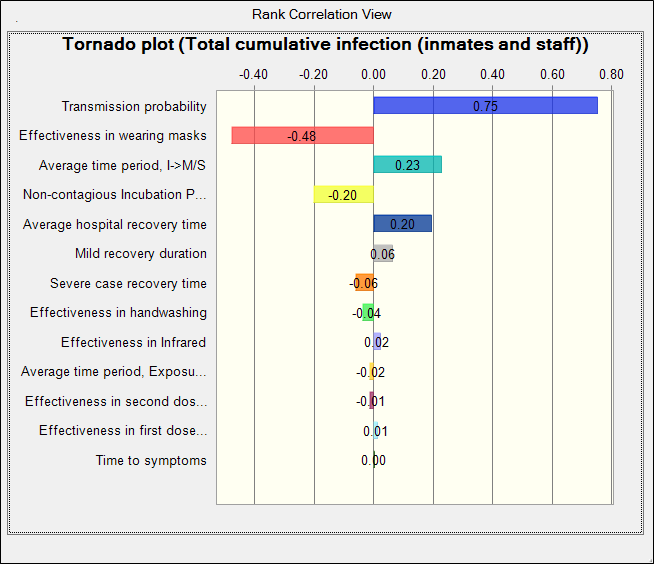 |

**Figure A.5: Number of new infections of COVID-19 among people in prison with the existing prevention strategies at the time of the outbreak (A) NSW, Australia and (B) Quebec, Canada, with 95% CI (blue dotted)**

| (A) | 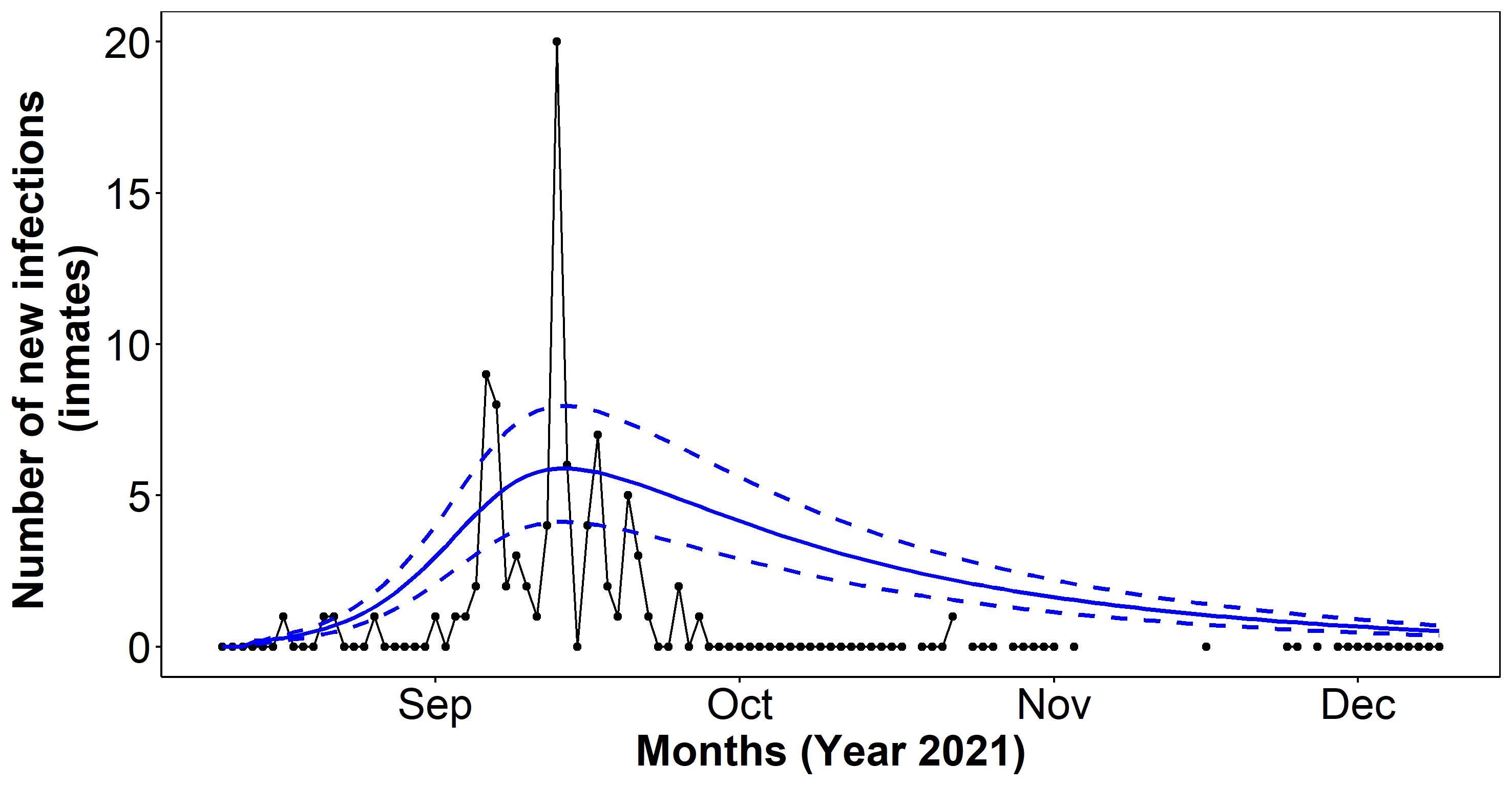 |
| --- | --- |
| (B) | 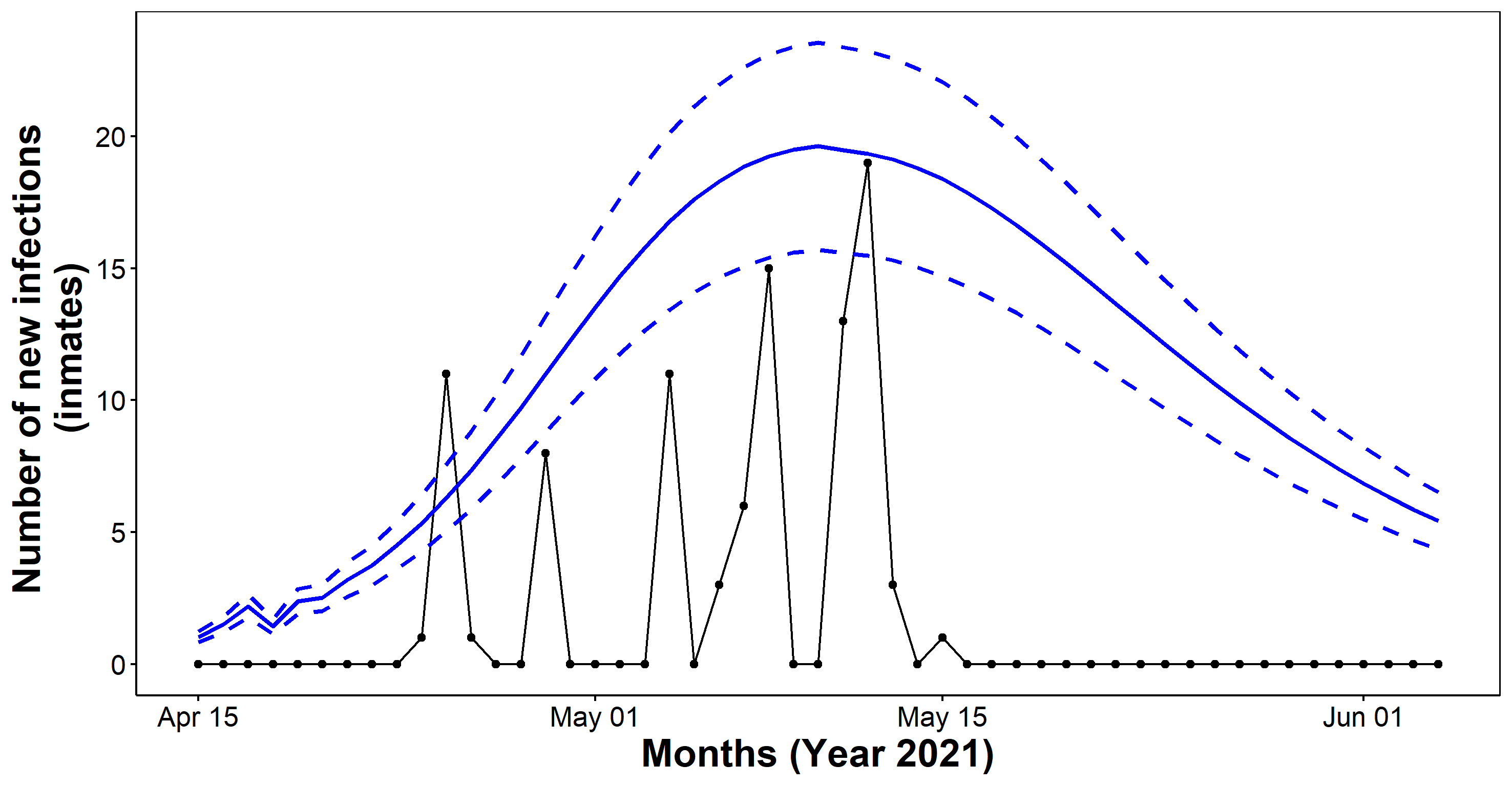 |
